# Minimally Manipulated Mesenchymal Stem Cells for the Treatment of Knee Osteoarthritis: A Systematic Review of Clinical Evidence

**DOI:** 10.1155/2019/1735242

**Published:** 2019-08-14

**Authors:** B. Di Matteo, F. Vandenbulcke, N. D. Vitale, F. Iacono, K. Ashmore, M. Marcacci, E. Kon

**Affiliations:** ^1^Department of Biomedical Sciences, Humanitas University, Via Manzoni 113, 20089 Rozzano, Milan, Italy; ^2^Humanitas Clinical and Research Center, Via Manzoni 56, 20089 Rozzano, Milan, Italy; ^3^First Moscow State Medical University, Sechenov University, Moscow, Russia

## Abstract

**Background:**

The use of laboratory-expanded mesenchymal stem cells (MSCs) is subject to several restrictions, resulting in “minimal manipulation” methods becoming the current most popular strategy to increase the use of MSCs in an orthopaedic practice. The aim of the present systematic review is to assess the clinical applications of “minimally” manipulated MSCs, either as bone marrow aspirate concentrate (BMAC) or as stromal vascular fraction (SVF), in the treatment of knee osteoarthritis (OA).

**Methods:**

A systematic review of three databases (PubMed, ScienceDirect, and Google Scholar) was performed using the following keywords: “Knee Osteoarthritis” with “(Bone marrow aspirate) OR (bone marrow concentrate)” or with “(adipose-derived mesenchymal stem cells) OR (adipose derived stromal cells) OR (stromal vascular fraction) OR (SVF)” as either keywords or MeSH terms. The reference lists of all retrieved articles were further reviewed for identification of potentially relevant studies.

**Results:**

Twenty-three papers were included in the final analysis (10 on BMAC and 13 on SVF). Of these, only 4 were randomized controlled trials (RCTs). Bias risk evaluation, performed using a modified Coleman score, revealed an overall poor quality of the studies. In terms of clinical application, despite the apparent safety of minimally manipulated MSCs and the short-term positive clinical outcomes associated with their use, clinicians reported different preparation and administration methods, ranging from single intra-articular injections to intraosseous applications to administration in combination with other surgical procedures.

**Conclusions:**

The available literature is undermined by both the lack of high-quality studies and the varied clinical settings and different protocols reported in the few RCTs presently published. This prevents any recommendation on the use of either product in a clinical practice. Nevertheless, the use of minimally manipulated MSCs (in the form of BMAC or SVF) has been shown to be safe and have some short-term beneficial effects.

## 1. Background

Among the large number of treatment possibilities for knee osteoarthritis (OA), novel regenerative medicine strategies are an area of growing interest [1]. This is especially the case in the challenging subset of younger OA patients, who have high functional demands yet limited indications for invasive surgical treatments. Nowadays, the issue has even extended to middle-aged active patients who increasingly expect to maintain a high activity level and postpone or avoid metal resurfacing. Despite the rising incidence of OA, no effective therapies have been shown to fully restore the original features and structure of the damaged articular surface. Furthermore, no “conservative” technique is able to ameliorate widespread damage to all articular tissues, which arises due to OA affecting the entire joint environment [2, 3].

Innovative therapies, ranging from platelet-derived growth factors (GFs) to cell-based treatments (sometimes combined with various biomaterials) [4, 5], have been proposed as potential solutions for these patients. Mesenchymal stem cells (MSCs) have emerged as a possible therapeutic option, thanks to their multilineage differentiation potential [6]. Mesenchymal cells are indeed able to differentiate into several cell types such as osteoblasts, chondrocytes, or adipocytes and have high plasticity, limited self-renewal capabilities, and immune-suppressive and anti-inflammatory actions [7]. Growth factors, cytokines, bioactive lipids, and microvesicles that are released from stem cells may also exert beneficial effects including angiopoietic and antiapoptotic actions. Studies have also highlighted the presence of MSCs in numerous adult tissues including adipose and muscle tissues, the dermis, periosteum, synovial membrane, synovial fluid, and articular cartilage [8].

Choosing the correct stem cell approach is imperative in achieving optimal results in regenerative medicine. Among the available sources of MSCs, two have received the greatest scientific attention: adipose-derived mesenchymal stem cells (ASCs) and the more studied bone marrow mesenchymal stem cells (BMSCs) [9, 10].

The clinical application of MSCs is strictly regulated especially in regard to cell expansion. In this case, despite being an autologous product, MSC products are considered “drugs,” and therefore, their use is extremely limited in a routine clinical setting, both in Europe and the USA [11]. This regulatory burden has incentivized clinicians to develop alternative strategies for MSC use, resulting in the currently crucial concept of “minimal manipulation.” If cells are not expanded but are rather manipulated within or “nearby” the operating room (OR), MSC application is easier and simpler, since the cells can be administered outside the boundaries of clinical trials (approved by local authorities and National Health Ministries). Following this reasoning, two main treatment modalities have emerged: bone marrow aspirate concentrate (BMAC) and adipose-derived stromal vascular fraction (SVF). BMAC is obtained via bone marrow needle aspiration (which can be performed on various sites but most commonly on the iliac crest) and subsequent concentration via dedicated centrifuges which can be transported and used directly within the OR [7, 12]. On the other hand, collection of adipose-derived SVF involves multiple steps: first, a liposuction is performed to obtain adipose tissue. In order to isolate the ASCs from the extracellular matrix (ECM), collagenase is added to the lipoaspirate [13]. Subsequently, the collagenase in the mixture is removed via a dilution method, which involves washing the lipid-enzyme mixture with normal saline solution, followed (in some cases) by sterile centrifugation. This results in the final SVF product, ready to be administered to the patient.

The aim of the present systematic review is to assess the clinical applications of “minimally manipulated” MSCs, both BMAC and SVF, in the treatment of knee OA.

## 2. Materials and Methods

### 2.1. Literature Search Strategy

We conducted this systematic review according to the Preferred Reporting Items for Systematic Reviews and Meta-Analyses (PRISMA) guidelines [14]. A systematic review of three medical electronic databases (PubMed, ScienceDirect, and Google Scholar) was performed by two independent authors (*N.D.V. and F.V.*) from 1998 to 20/10/2018. To achieve maximum search strategy sensitivity, we combined the terms “Knee Osteoarthritis” or “Knee OA” with “(Bone marrow aspirate) OR (bone marrow concentrate)” or with “(adipose-derived mesenchymal stem cells) OR (adipose derived stromal cells) OR (stromal vascular fraction) OR (SVF)” as either keywords or MeSH terms. The reference lists of all retrieved articles were further reviewed for identification of potentially relevant studies and assessed using the inclusion and exclusion criteria stated below.

**Figure 1:**
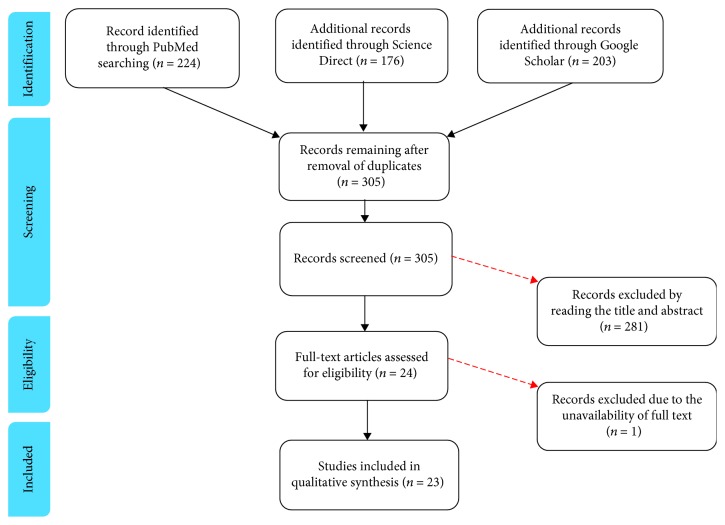
PRISMA (Preferred Reporting Items for Systematic Reviews and Meta-Analysis) flowchart of the systematic literature review.

Eligible studies for the present systematic review included those dealing with the intra-articular use of minimally manipulated BMAC or SVF in knee OA. The initial title and abstract screening was made using the following inclusion criteria: studies of any level of evidence, written in English, and reporting clinical results following the intra-articular application of either BMAC or SVF as a treatment approach for OA. Conversely, articles dealing with expanded or otherwise manipulated mesenchymal stem cells were excluded. Papers where MSCs were applied for other clinical indications (such as focal cartilage defects) were also excluded. We further excluded all duplicate articles, articles from non-peer-reviewed journals, or articles lacking access to the full text. Conference presentations, narrative reviews, editorials, and expert opinions were also excluded. A PRISMA [14] flowchart of the selection and screening method is provided in Figure 1.

All data were extracted from article texts, tables, and figures. Two investigators independently reviewed each article (*F.V. and N.D.V.*). Discrepancies between the two reviewers were resolved by discussion and consensus. The final results were reviewed by senior investigators (*B.D.M. and E.K.*). Risk of bias assessment of the included articles was done following the Coleman methodology score modified by Kon et al. [15]. The assessment was independently performed by 2 authors (*F.V.* and *N.D.V*.). Any discrepancy was discussed with and resolved by the senior investigators, who made the final judgement.

## 3. Results

According to the aforementioned inclusion and exclusion criteria, 23 articles were ultimately included in the present review. Relevant data is detailed in Tables 1 and 2. Thirteen of the included articles involved the use of SVF; the other ten involved the use of BMAC.

### 3.1. BMAC

#### 3.1.1. Application Methods and Quality Assessment of the Available Literature

The results of the studies' quality assessment performed with the Coleman methodology score modified by Kon et al. are detailed in Table 3. The average score was 37.4 out of 100, thus showing overall poor methodology in the available literature.

The ten studies investigating BMAC involved 1710 knees from 1386 patients affected by OA. The concentrate was injected intra-articularly in 8 studies [16–23] and within the tibial and femoral subchondral bones in two other papers [24, 25]. The aspirated bone marrow was centrifuged without any other manipulation, expansion, or culture before administering it to the patients.

Regarding the therapeutic protocol, five papers dealt with the administration of BMAC alone: two of these involved subchondral BMAC injections [24, 25], whilst in the remaining three, BMAC was injected intra-articularly [16–18]. Of these last three papers, one study involved the administration of 4 sequential injections within a 3-month timespan [17]. Other authors administered combined BMAC and PRP within the same session [19, 21, 22] or administered PRP alone after a certain period as a booster injection [20]. Finally, two authors injected BMAC in association with adipose tissue [22, 23].

In regard to the clinical outcome, the majority of authors employed reliable clinical scores (such as those derived from WOMAC, IKDC, KOOS, or KSS), whereas only a few authors used less known questionnaires, improvement rating scores, or patient satisfaction [17, 20–22]. All studies assessed pain through a visual or numerical score such as VAS or NPS, except for two authors [18, 25] who included WOMAC and KSS questionnaires, which have sections on patient pain. Finally, three authors performed MRI prior to and following the procedure [19, 24, 25].

Concerning the study design of the 10 included articles, only 2 were randomized controlled trials (RCTs) [19, 25], 1 was a prospective pilot trial [24], and the rest were retrospective. Of the latter group, two were comparative studies: one compared two groups of patients receiving BMAC at different cell concentrations [21], whilst the other compared a group treated with BMAC+PRP to a group treated with BMAC+PRP+adipose tissue [22]. Although two RCTs were identified, in both cases, the control group was the contralateral knee, which introduces into data gathering and interpretation [19, 25].

#### 3.1.2. Clinical Findings

The main finding of the reviewed papers was a significant improvement in pain and function in almost all cohorts. However, no superiority has been demonstrated over standard treatments such as viscosupplementation or corticosteroids. Shapiro et al. [19] performed the only placebo-controlled RCT available, and they found no statistically significant differences between BMAC and saline injections. Moreover, studies on MRI reported conflicting outcomes. Whilst Shapiro et al. [19] found no evidence of cartilage regeneration on MRI scans, in their pilot trial, Vad et al. [24] detected an increase in extracellular matrix thickness by an average of 14%, especially in younger patients. MRI outcomes were dramatically better in osteonecrotic patients where bone and cartilage repair was observed, with a reduction in bone marrow lesion size by 40% [25]. Nevertheless, it seems that a higher BMAC cell concentration is associated with a significantly better outcome [21]. Furthermore, Shaw et al. suggested that multiple injections could be more effective than a single one since each subsequent treatment provided additional benefit to the patients [17]. On the other hand, the administration of adipose tissue in combination with BMAC showed no benefit in comparison to BMAC without adipose tissue [22].

### 3.2. SVF

#### 3.2.1. Application Methods and Quality Assessment of the Available Literature

The mean Coleman methodology score (modified by Kon et al.) of the 13 studies was 47 out of 100 (Table 4), slightly better than that of BMAC studies but still insufficient to define the available evidence as “methodologically” robust.

Several different therapeutic approaches were employed in the studies regarding SVF. Only 4 studies [26–29] included in this review analysed the results of knee intra-articular SVF concentrate injections with no other additional treatments. SVF was administered intra-articularly in another 3 studies [30–32], but this was injected in combination with platelet-rich plasma (PRP). The majority of the authors, however, injected SVF following an arthroscopic surgical procedure, with 3 of them administering SVF after arthroscopic debridement or lavage [33–35]. Conversely, Koh et al. [36] performed arthroscopic lavage prior to an injection of SVF and PRP combined. Nguyen et al. [37] injected SVF suspended in PRP after arthroscopic microfracture of the osteoarthritic bone. Only one study [32] reported the use of arthrotomic surgery in the therapeutic protocol, with SVF+PRP being injected after performing open-wedge high tibial osteotomy. Regarding clinical outcomes, only 1 [26] of the 13 papers concerning SVF did not include at least one knee-specific patient-reported score as calculated through validated questionnaires, namely, WOMAC, IKDC, KOOS, Lysholm score, or Tegner activity scale. None of these scores were employed in the study performed by Hudetz et al. [26], which was conversely the only study in which laboratory measurements were taken into consideration. The authors examined the effects of autologous microfragmented fat tissue injection on the synthesis of proteoglycans within the synovial fluid. Immunohistochemical analysis was only reported in Roato et al.'s study [29]. In almost all of the studies, the VAS scale was used to quantify osteoarthritic pain relief following SVF administration. Changes in MRI images following the therapeutic procedure were taken into account in 7 studies [26, 29–31, 33, 37, 38]. Second-look arthroscopy of the affected joint was instead performed in 4 different papers [34–36]. Our review of the literature revealed a paucity of randomized controlled trials concerning the clinical application of SVF in the treatment of OA, with only 2 trials out of 13 being RCTs [28, 33]. Nine papers were prospective clinical studies, with 3 of them being comparative [34, 35, 37]. The rest were retrospective studies.

#### 3.2.2. Clinical Findings

The review of the literature highlighted some important concepts about SVF and its clinical application. Firstly, regarding its safety in the treatment of OA, no serious adverse events have been described in any of the 13 articles dealing with this topic, with only mild swelling and pain being reported in very few patients within the first few days following the therapeutic procedure. In regard to donor site morbidity, liposuction was very well tolerated by patients mainly due to the limited amount of tissue harvested. Secondly, SVF administration has shown positive clinical outcomes in the studies reviewed. A significant improvement in terms of range of movement, pain, and articular function during daily activities (measured via varied and commonly used validated questionnaires such as WOMAC, IKDC, KOOS, Lysholm score, Tegner activity scale, and VAS pain scale) was reported in all 13 studies at the last follow-up. Thirdly, regarding MRI, most of the papers [26, 30, 31, 33, 38] (including the RCT by Hong), described positive changes in the imaging outcomes, reporting signs of partial regeneration of the articular cartilage. Similarly, Nguyen et al. [37] described significantly less bone marrow edema in patients receiving PRP+SVF treatment following microfracture compared to the control group (microfracture only). Nevertheless, Roato et al. [29] did not report a significant difference between MRI scores before SVF treatment compared to MRI scores at 18 months, as measured via the use of Outerbridge scores, a quantitative parameter (ranging from 1 to 4) determining the OA grade of the affected knee. The same study also took into consideration the histological outcome of two patients who left the study after 12 and 14 months, respectively, to undergo knee arthroplasty. Knee biopsies were performed and compared to those collected from OA patients (matched for age, sex, and OA grading) who underwent arthroplasty alone. In both the joints previously treated with SVF, the authors detected the presence of new tissue formation starting from the subchondral end of the osteochondral lesion, whereas this type of neotissue formation was undetectable in those that did not receive an SVF injection.

These encouraging results concerning nonexpanded adipose tissue injections are however weakened by the presence of several biases in the studies' therapeutic protocols. In fact, in only 4 out of 13 studies [26–29] was adipose tissue solely administered. In the remaining papers, concomitant surgery or administration of other injective therapies (viscosupplementation, PRP) was performed, obviously confusing the obtained outcomes and making them difficult to compare.

## 4. Discussion

The main finding of the present systematic review is that the available literature concerning the use of BMAC and SVF for knee OA is characterized by a lack of sound methodologies, as represented by the Coleman methodology scores, with a paucity of RCTs being presently published, thus preventing us from making solid conclusions on the real therapeutic potential of these novel methods compared with others.

Currently, there are several clear issues in the scientific literature. Although clinical results seem positive, when assessing the risk of bias, it becomes apparent that most of the studies present are of low quality and lack relevant methodologies, as detailed in Table 3. Average Coleman scores (modified by Kon et al.) were exceedingly poor mainly due to the mean follow-up being short and half of the included studies being retrospective, with only four papers being RCTs [19, 25, 28, 33] and eight being prospective studies. Furthermore, in many papers, the inadequate patient selection process introduced another source of bias, since inclusion and exclusion criteria were seldom reported, the recruitment rate was not stated, and a flowchart of the selection process was often unavailable. Although all the authors adequately described the procedure, in most of the studies, many patients underwent concomitant surgery, such as arthroscopic debridement, microfracture, or high tibial osteotomy, thus preventing a clear understanding of the real contribution and clinical potential of these stem cell-based products. Furthermore, whilst outcome criteria were always clearly defined and mostly demonstrated good reliability and sensitivity, the procedures for assessing the clinical outcome were not fully elucidated. In addition, MRI outcomes were only reported in 11 studies (10/23 43,5%).

The overall lack of sounding methodologies, along with the inherent paucity of RCTs available, is not an uncommon observation in the field of biologic therapies for OA. If we look at the recent past, in many cases, biotechnologies have been released onto the market without enough proof of evidence: the paradigmatic example is platelet-rich plasma (PRP), which “invaded” clinical practice on the basis of huge media exposure rather than solid scientific evidence [39]. One reason for this lies within the possibility of taking regulatory shortcuts such as the 510(k) exemption [40], based on the fact that new medical devices “substantially equivalent” to others already present on the market can skip the standard FDA approval process. This led to a proliferation of commercially available kits for PRP production, and in a few years, the market was saturated by many different preparation systems giving substantially different outputs in terms of biological features, thus inhibiting a “standardization” of PRP therapy for the treatment of OA or other musculoskeletal conditions. The same risk is now impending on stem cell-based technologies, especially on the so-called “minimally manipulated” products, which are not affected by the regulatory burden currently imposed on products requiring cell expansion in lab. Consequently, BMAC and SVF have emerged as the easiest way to employ MSCs in the clinical practice, since these products can be rapidly harvested directly within the OR and be immediately administered to patients in disparate ways, such as simple intra-articular injections (sometimes in association with other carriers like PRP or hyaluronic acid), as intraosseous injection at the bone-cartilage interface, as augmentation for various scaffolds, and as a topical application on focal defects [25, 34, 38]. Whilst the wide range of potential applications is fascinating, it is also responsible for the current lack of clear indications on the best method of therapeutic administration. The concurrent use of other biological agents or biomaterials or the administration of these cellular products following “conventional” surgical procedures introduces additional confounding elements, thus preventing a fair comparison of the studies performed so far. Even the few RCTs published present some relevant biases, mainly due to the fact that in most of them patients were treated bilaterally [19, 25, 33], which is not the ideal condition to assess the efficacy of a treatment considering that patients cannot evaluate one knee independently from the other. This results in a bias that is very difficult to account for even when using dedicated statistical tests [41]. Basic questions such as the number of cells administered, the optimal number of injections to achieve the best therapeutic effect, and the superiority of one preparation method over another still remain unanswered. Despite the attempts presently published, the literature is still lacking well-designed trials to address these issues. Similarly, it has been impossible to establish which, or whether, one of the two sources, either bone marrow or adipose tissue, provides better results. Whilst it has been shown that the immunophenotypes of BMSCs and ASCs are more than 90% identical [42, 43], they still exhibit a number of distinct characteristics, for example, in their cell surface markers, differentiation potentials, and in their distribution within the body. In vitro analysis revealed that the great advantage of SVF is their abundance: when compared to 100 ml of bone marrow aspirate, up to 300-fold more stem cells can be obtained from 100 g of adipose tissue [44, 45]. Regardless, since a dose-effect correlation has not been clearly demonstrated, this advantage still remains theoretical. Lastly, the issue of interhuman variability, which plays a central role in the success of these biological therapies (since a particular “patient's profile” could respond better to a specific biologic stimulus compared to another), has not yet been faced. This exacerbates the need for more research, dedicated to understanding the unique features of a specific stem cell-based product in order to target the unique features of the recipient joint of a particular patient.

From a “surgical” point of view, bone marrow harvesting and lipoaspiration are both simple procedures with minimal side effects (with the latter being perhaps slightly more serious due to the associated risks of hematoma and pain at the site of lipoaspiration). Currently, the choice between bone marrow and fat tissue is purely based on the surgeon's preference and experience and, keeping commercial issues in mind, on the different availability of preparation kits in different countries. However, in recent years, more and more industries have been releasing their proprietary kits for BMAC and SVF preparation with new methods still being developed, such as the use of mechanical microfragmentation of the adipose tissue to obtain an “adipose graft” containing intact stromal vascular niches with MSCs and other cells involved in the modulation of joint homeostasis [46]. Whilst the increase in the number of preparation kits on the market has contributed to a partial reduction of costs over time, it has also raised the risk of releasing products with dubious performances and without sufficient scientific data certifying their capability of concentrating MSCs in a “minimal manipulation”-compliant approach. In addition, the overexposure of biological products for OA has led to their rapid growth (both within the market of “dedicated devices” and within clinical settings), without the support of robust evidence. Expanding the number of available treatment options for patients affected by knee OA does not always mean improving the standard of care, especially when there is a lack of comparative trials assessing the effectiveness of a novel treatment compared to established ones.

Presently, stem cell-based products undoubtedly represent an expensive technology, whose costs still cannot be sustained by National Health Systems, and (due to the lack of robust data on their effectiveness) cannot be endorsed as a “routine” treatment for knee cartilage degeneration. From a clinical point of view, despite the aforementioned methodological limitations, the use of BMAC and SVF for the treatment of knee OA seems safe and able to provide positive clinical outcomes, thus potentially offering a new minimally invasive therapeutic option for patients who are not eligible for more invasive approaches. Nevertheless, their promising, short-term results must be both confirmed in the long-term and compared to those of established treatments. Until then, the use of minimally manipulated MSCs, either BMAC or SVF, represents a therapeutic option that must be carefully and thoroughly discussed between the physician and the patient, especially when it is proposed as a first-line therapeutic approach to avoid more invasive solutions, rather than a “salvage procedure.”

## 5. Conclusion

The available literature is undermined by both the lack of high-quality studies and the varied clinical settings and different protocols reported in the small number of RCTs published. This prevents any recommendation on the use of either product in the clinical practice. Nevertheless, focusing on clinical results, BMAC and SVF have been shown to be safe and to have some short-term beneficial effect on the treatment of knee cartilage degeneration. Currently, there is no evidence on the superiority of either bone marrow or adipose tissue as a source of minimally manipulated MSCs.

## Figures and Tables

**Table 1:** Clinical studies regarding the use of bone marrow aspirate concentrate (BMAC) in the treatment of knee osteoarthritis.

Publication	Study design	Disease	Therapeutic protocol	Outcome	Patient characteristic	F-up	Main findings
Hernigou et al. [25]	RCT (TKA on contralateral knee)	Bilateral OA secondary to severe ON related to corticosteroids	BMAC graft percutaneously injected to the subchondrium of the femur and tibia vs. TKA on contralateral knee	MRI, radiographs, bone marrow lesion volume, Knee society score	30 (30 BMAC, 30 TKA) *Age:* 18-41 *Sex:* M-F: 12-18 *K-L:* IV	8-16 years (mean: 12)	Decrease in ON size by 40%. Cartilage and bone repair observed. Outcome was not statistically significantly different between BMAC and TKA. The majority of patients preferred BMAC.
Themistocleous et al. [16]	Retrospective	OA	BMAC injection alone	NPS and OKS	121 *Age:* 70 (50-85) *Sex:* M-F: 36-85 *K-L:* III-IV	11 months (range 6-30)	Significant improvement of both knee pain and function.
Shaw et al. [17]	Retrospective	OA	4 sequential BMAC injections in 3 months	Resting/active NPS, overall percentage improvement and LEFS	15 (20 knees) *Age:* 67.7 (7.9) *Sex:* M-F: 5-10 *K-L:* N/A	24 days from last injection	Significant improvement of both knee pain and function. The additional benefit with each subsequent treatment may suggest that multiple injections are more effective than a single one.
Rodriguez-Fontan et al. [18]	Retrospective	OA	12 ml BMAC injection alone	WOMAC Satisfaction rate	19: 10 knees and 15 hips *Age:* mean 58 (30-80) *Sex:* M-F: 3-16 *K-L: I-II*	6-24 months	Significantly improved WOMAC score; no significant difference between six-month and latest follow-up scores. Variable satisfaction rate (63.2% yes, 36.8% no).
Shapiro et al. [19]	Single-blind RCT (placebo on contralateral knee)	Bilateral OA	BMAC+platelet-poor plasma (PRP) vs. saline injection	VAS, ICOAP, and algometer	25 (25 vs. 25 knees) *Age:* median 60 (42-68) *Sex:* M-F: 7-18 *K-L:* I-III	12 months	Significant improvement in pain and quality of life. No superiority to saline injection. No evidence for cartilage regeneration on MRI (T2 mapping).
Sampson et al. [20]	Retrospective	OA	BMAC injection followed by a PRP booster injection at approximately 8 weeks	VAS and global patient satisfaction	73 (100 knees) *Age:* range 23-79 *Sex:* N/A *K-L:* III-IV	5 months	Significant improvement of knee pain. High level of patient satisfaction.
Vad et al. [24]	Pilot trial	OA	Injection of tibial BMAC to the femoral and tibial chondral-bone interface and intra-articular knee joint space via the PeCaBoo delivery system	MRI, WOMAC, participant-reportednumeric pain rating scale	10 *Age:* 63.5 (52-73) *Sex:* M-L: 4-6 *K-L:* III-IV	13-15 months (mean: 14)	Significant improvement in WOMAC and NRS scores. MRI displayed an increase in extracellular matrix thickness by an average of 14%. Improvements were more substantial for patients younger than 63.5 years old.
Centeno et al. [21]	Comparative retrospective (registry data) Group A vs. B	OA	(A) <4 × 10^8^ cells BMAC+PRP+PL (B) >4 × 10^8^ cells BMAC+PRP+PL	NPS, LEFS, IKDC, improvement rating score	373 (424 knees): (224 vs. 185) *Age:* 54.5 vs. 50.2 *Sex:* M-F:143-81/140-45 *K-L:* I-IV	3-15 months	Significant improvement of both knee pain and function. Significantly higher pain reduction in patients treated with BMAC with high cell content.
Centeno et al. [22]	Comparative retrospective (registry data) Group A vs. B	OA	(A) BMAC+PRP vs. (B) BMAC+PRP+adipose tissue	NPS, LEFS, improvement rating score	681 (840 knees): (616 vs. 224) *Age:* 54.3 vs. 59.9 *Sex:* M-F: 397 : 219/119 : 105 *K-L:* I-IV	6-10 months	Significant improvement of both knee pain and function. No detectible benefit with the addition of an adipose graft to the BMAC.
Kim et al. [23]	Retrospective	OA	BMAC+adipose tissue inj.+arthroscopic debridement (6), microfractures (5), and HTO (1)	VAS, IKDC, SF-36, KOOS, Lysholm	41 (75 knees) *Age:* 60.7 (53–80) *Sex:* M-F: 17-24 *K-L:* I-IV	8.7 months	Significant improvement of both knee pain and function. Better outcomes in early to moderate phases.

**Table 2:** Clinical studies regarding the use of stromal vascular fraction (SVF) in the treatment of knee osteoarthritis.

Publication	Study design	Disease	Therapeutic protocol	Outcome	Patient characteristic	F-up	Main findings
Jones et al. [28]	RCT (NCT03242707)	OA	Comparative study: ultrasound-guided, intra-articular injection of autologous adipose tissue vs. HA	WOMAC, PROMIS questionnaire, synovial fluid analysis, sway velocity assessment	54 (27 vs. 27) Age: N/A Sex: M-F: N/A K-L: N/A	6 months	Ongoing.
Roato et al. [29]	Prospective	OA	Following diagnostic arthroscopy, 35 ml of concentrated adipose tissue was injected intra-articularly	WOMAC, VAS, MRI, immunohistochemistry of 2 knees	20 *Age:* 59.6 *Sex:* M-F: 9-11 *K-L:* I-III	18 months	Whilst both WOMAC and VAS scores improved significantly, WOMAC scores showed progressively better outcomes. MRI Outerbridge grade did not show significant changes. Immunohistochemistry displayed new tissue growth.
Hong et al. [33]	Double-blind RCT (HA in contralateral knee)	Bilateral OA	Comparative study: arthroscopic debridement followed by intra-articular SVF injection vs. HA injection in the contralateral knee	VAS, WOMAC, ROM, whole-organ MRI score, MRI observation of cartilage repair tissue	16 (32 knees): (16 vs. 16) *Age:* 18-70 years *Sex:* M-F: *K-L:* II-III	12 months	VAS and WOMAC scores and knee ROM improved significantly for both groups, but these improvements were not long lasting in the control group. MRI analysis showed significantly increased cartilage repair in the SVF group compared to the control.
Hudetz et al. [26]	Prospective	OA	Microfragmented adipose tissue injection	VAS, radiographs, dGEMRIC MRI, IgG isolation from plasma and synovial fluid	17 (32 knees) *Age:* 40-85 *Sex:* M-F: 12-5 *K-L:* III-IV	12 months	Significant decrease in VAS scores. No change in IgG glycome composition. dGEMRIC MRI analysis displayed increase in proteoglycan content within the ECM.
Bansal et al. [30]	Prospective (phase I) NCT03089762	OA	SVF+PRP injection	WOMAC, 6-minute walking distance, MRI	10 (13 knees) *Age:* ≥50 *Sex:* N/A *K-L:* I-II	24 months	Significant improvement of WOMAC scores and 6-minute walking distance. MRI showed increase in cartilage thickness in all but 2 patients. All patients are satisfied with therapy.
Yokota et al. [27]	Prospective	OA	Intra-articular injection of SVF	VAS, WOMAC, Japanese Knee Osteoarthritis Measure (JKOM)	13 (26 knees) Age: 74.5 Sex: M-F: 2-11 *K-L:* III-IV	6 months	All VAS, WOMAC, and JKOM scores improved significantly at the 6-month (last) follow-up.
Nguyen et al. [37]	Comparative prospective	OA	Comparative study: arthroscopic microfracture (AM) and SVF+PRP injection vs. AM alone	WOMAC, VAS, and Lysholm scores, MRI, knee joint function	30 (15 vs. 15) *Age:* 58.60 vs. 58.20 *Sex:* M-F: 3-12vs. 3-12 *K-L:* II-III	18 months	WOMAC, Lysholm, and VAS scores improved for both groups up to 12 months, but at 18 months, the SVF group was significantly better than the control group. At 12 months, the SVF group displayed significantly less bone marrow edema than the control group.
Koh et al. [36]	Prospective	OA	Following arthroscopic lavage is intra-articular injection of SVF+PRP to the most severe cartilaginous defects	Lysholm, VAS, and KOOS scores, radiographs, 2nd-lookarthroscopy	30 *Age:* ≥60 *Sex:* M-F: 5-25 *K-L:* II-III	24 months	Lysholm, VAS, and KOOS scores all improved significantly. Scores increased at the second year compared to the first year of follow-up. Second-look arthroscopy determined the majority of knees as positive or better.
Kim et al. [34]	Prospective comparative	OA	Comparative study: following arthroscopic debridement, group 1 received an intra-articular injection of SVF; group 2 received an intra-articular injection of SVF+fibrin glue as a scaffold	IKDC, Tegner, 2nd-lookarthroscopic ICRS grading	54 (56 knees): 37 vs. 17 *Age:* 57.5 vs. 57.7 *Sex:* M-F: 14-23vs. 8-9 *K-L:* I-II	29.2 vs. 27.3 months	IKDC and Tegner activity scores significantly improved in both groups but showed no significant difference. Statistical significance between the two groups was observed in ICRS grades, with the SVF group being more positive. A higher BMI resulted in less positive outcomes.
Koh et al. [32]	Prospective comparative	OA	Comparative study: intra-articularinjection of SVF+PRP vs. only PRP prior to performing open-wedge high tibial osteotomy	Lysholm, KOOS, VAS, and femorotibial angle. Arthroscopic evaluation	44 (23 vs. 21) *Age:* 52.3 vs. 54.2 *Sex:* M-F: 6-17vs. 5-16 *K-L:* I-III	24-25 months (mean: 24.4)	Lysholm, VAS score, and KOOS improved statistically in both groups. KOOS improvements were statistically greater in the SVF group. No difference in the preoperative and postoperative femorotibial angles. SVF group displayed greater fibrocartilage coverage.
Koh et al. [35]	Retrospective	OA	Arthroscopic debridement+administration of SVF to articular chondral lesions	IKDC score and Tegner activity scale, 2nd-look arthroscopic ICRS grading	35 (37 knees) *Age:* 48-69 *Sex:* M-F: 14-21 *K-L:* I-II	24-34 months (mean: 26.5)	IKDC and Tegner activity scores significantly improved. Patients reported high satisfaction scores. It was noted that a higher BMI resulted in less positive outcomes.
Bui et al. [31]	Prospective	OA	Intra-articular SVF+PRP injection	VAS and Lysholm scores, MRI	21 *Age:* ≤18 *Sex:* N/A *K-L:* II-II	6 months	Statistically significant improvement in VAS and Lysholm scores. MRI analysis showed partial regeneration and thickening of articular cartilage.
Koh et al. [38]	Retrospective	OA	Infrapatellar SVF+PRP, intra-articularinjection+weekly PRP injection for 2 weeks	Whole-organ MRI, WOMAC, VAS, and Lysholm scores	18 *Age:* 41-69 *Sex:* M-F: 6-12 *K-L:* III-IV	24-26 months (mean: 24.3)	Significant decrease of WOMAC, VAS, and Lysholm scores. Significant decrease of whole-organ MRI scores. Extent of improvement was directly correlated with the amount of MSCs injected.

NPS: numerical pain scale; OKS: Oxford Knee Score; LEFS: Lower Extremity Functionality Score; VAS: visual analog scale; OARSI: Osteoarthritis Research Society International; ICOAP: Intermittent and Constant Osteoarthritis Pain; WOMAC: Western Ontario and McMaster Universities Arthritis Index; TKA: total knee arthroplasty; IKDC: International Knee Documentation Committee; KOOS: Knee injury and Osteoarthritis Outcome Score; MACI: Matrix-induced Autologous Chondrocyte Implantation; ROM: range of motion; ICRS: International Cartilage Regeneration & Joint Preservation Society; PeCaBoo: percutaneous cartilage-bone interface optimization system; dGEMRIC: delayed gadolinium-enhanced magnetic resonance imaging of cartilage.

**Table 3:** BMAC study quality assessment with the Coleman methodology score modified by Kon et al.

	Study	TOT	Study size	Mean f-up	Different surg proc	Type of study	Surg proc description	Postop rehab	MRI outcome	Histological outcome	Outcome criteria	Outcome assessment	Selection process
Bone marrow aspirate concentrate	Hernigou et al. [25]	70	7	10	10	15	5	5	10	0	5	3	0
	Themistocleous et al. [16]	44	10	0	10	0	5	5	0	0	5	3	6
	Shaw et al. [17]	23	0	0	10	0	5	0	0	0	5	3	0
	Rodriguez-Fontan et al. [18]	30	0	2	10	0	5	2	0	0	5	3	3
	Shapiro et al. [19]	51	7	2	4	15	5	0	10	0	2	3	3
	Sampson et al. [20]	26	10	0	4	0	5	2	0	0	2	0	3
	Vad et al. [24]	49	0	2	10	10	5	0	10	0	5	7	0
	Centeno et al. [21]	26	10	0	4	0	5	2	0	0	2	3	0
	Centeno et al. [22]	23	10	0	4	0	5	2	0	0	2	0	0
	Kim et al. [23]	32	10	0	4	0	5	5	0	0	5	3	0

**Table 4:** SVF study quality assessment with the Coleman methodology score modified by Kon et al.

	Study	TOT	Study size	Mean f-up	Different surg proc	Type of study	Surg proc description	Postop rehab	MRI outcome	Histological outcome	Outcome criteria	Assessment of clinical outcome	Selection process
Adipose-derived stem cells	Jones et al. [28]	54	7	0	10	15	5	0	0	0	5	9	3
	Roato et al. [29]	67	0	2	10	10	5	5	10	10	5	7	3
	Hong et al. [33]	56	4	2	4	15	5	5	10	0	5	3	3
	Hudetz et al. [26]	53	4	2	10	10	5	0	10	0	5	7	0
	Bansal et al. [30]	45	0	5	4	10	5	0	10	0	5	3	3
	Yokota et al. [27]	34	0	0	10	10	5	2	0	0	2	5	0
	Nguyen et al. [37]	51	4	2	4	10	5	5	10	0	5	3	3
	Koh et al. [36]	36	4	5	4	10	5	0	0	0	5	3	0
	Kim et al. [34]	47	7	5	4	10	5	5	0	0	5	3	3
	Koh et al. [32]	45	7	5	4	10	5	0	0	0	5	3	6
	Koh et al. [35]	40	10	5	4	0	5	5	0	0	5	3	3
	Pham 2014 Biomed Res	44	4	0	4	10	5	0	10	0	5	3	3
	Koh et al. [38]	40	4	5	4	0	5	2	10	0	5	2	3
